# Next-generation phenomics for the Tree of Life

**DOI:** 10.1371/currents.tol.085c713acafc8711b2ff7010a4b03733

**Published:** 2013-06-26

**Authors:** J. Gordon Burleigh, Kenzley Alphonse, Andrew J Alverson, Holly M Bik, Carrine Blank, Andrea L Cirranello, Hong Cui, Marymegan Daly, Thomas G Dietterich, Gail Gasparich, Jed Irvine, Matthew Julius, Seth Kaufman, Edith Law, Jing Liu, Lisa Moore, Maureen A O'Leary, Maria Passarotti, Sonali Ranade, Nancy B Simmons, Dennis W. Stevenson, Robert W Thacker, Edward C Theriot, Sinisa Todorovic, Paúl M. Velazco, Ramona L Walls, Joanna M Wolfe, Mengjie Yu

**Affiliations:** University of Florida; KenX Technology, Medford, New York; Department of Biological Sciences, University of Arkansas; UC Davis Genome Center; University of Montana; Department of Mammalogy, American Museum of Natural History; School of Information Resources & Library Science, University of Arizona; Department of Evolution, Ecology, and Organismal Biology, The Ohio State University; School of Electrical Engineering and Computer Science, Oregon State University; Department of Biological Sciences, Towson University; School of Electrical Engineering and Computer Science, Oregon State University; Department of Biological Sciences, St. Cloud State University; Whirl-i-Gig, Greenport, New York; Center for Research on Computation and Society, School of Engineering and Applied Sciences, Harvard University; Department of Biology, University of Florida; ProfessorUniversity of Southern Maine; Department of Anatomical Sciences, Stony Brook University; Whirl-i-Gig, Greenport, New York; School of Information Resources & Library Science, University of Arizona; Department of Mammalogy, American Museum of Natural History; The New York Botanical Garden; The University of Texas at Austin, Texas Natural Science Center; School of Electrical Engineering and Computer Science, Oregon State University; Department of Mammalogy, American Museum of Natural HistoryAmerican Museum of Natural History; iPlant CollaborativeUniversity of Arizona; American Museum of Natural History; The University of Texas at Austin, Texas Natural Science Center

## Abstract

The phenotype represents a critical interface between the genome and the environment in which organisms live and evolve. Phenotypic characters also are a rich source of biodiversity data for tree building, and they enable scientists to reconstruct the evolutionary history of organisms, including most fossil taxa, for which genetic data are unavailable. Therefore, phenotypic data are necessary for building a comprehensive Tree of Life. In contrast to recent advances in molecular sequencing, which has become faster and cheaper through recent technological advances, phenotypic data collection remains often prohibitively slow and expensive. The next-generation phenomics project is a collaborative, multidisciplinary effort to leverage advances in image analysis, crowdsourcing, and natural language processing to develop and implement novel approaches for discovering and scoring the phenome, the collection of phentotypic characters for a species. This research represents a new approach to data collection that has the potential to transform phylogenetics research and to enable rapid advances in constructing the Tree of Life. Our goal is to assemble large phenomic datasets built using new methods and to provide the public and scientific community with tools for phenomic data assembly that will enable rapid and automated study of phenotypes across the Tree of Life.

## Introduction

Biologists and non-biologists alike relate intuitively to the natural world and its underlying scientific principles through phenotypes. Phenomic data (e.g., morphology, behavior, physiology and other phenotypic traits) are also fundamental to inferring evolutionary histories 1 2 3 4 5 and enable systematists to integrate fossil taxa directly into phylogenetic trees. The placement of extinct taxa in phylogenies is essential for understanding patterns of diversification^6^ ^7^ and can greatly improve our understanding of trait evolution^8^. Thus, constructing the Tree of Life (ToL), representing the evolutionary history of all organisms, and understanding the patterns and processes of evolution are impossible without phenomic data. Technological advances, such as next-eneration sequencing, have dramatically increased the scale and decreased the cost of molecular sequencing, transforming the field of molecular phylogenetics. By contrast, matrices of phenotypic characters for phylogenetic analysis are still largely generated manually using methods that have not changed significantly for decades. This situation represents a major bottleneck for assembling the ToL and for evolutionary biology research in general. This problem motivated the organization of the next-generation phenomics project.

Biologists working with phenotypic data from taxa across the ToL are challenged by the difficulty of discovering and scoring characters, generating images that describe characters, and annotating and extracting phylogenetically informative data from legacy taxonomic and natural history literature. The next-generation phenomics project is leveraging innovations from computer science and engineering to build new tools to assemble phenomic character-by-taxon matrices cheaply and efficiently. Our project focuses on three distinct areas: 1) computer vision approaches to discover and score characters; 2) crowdsourcing approaches to increase the speed of scoring matrices and generating datasets enriched with labeled anatomical images; and 3) natural language processing approaches to extract character data to build matrices from legacy taxonomic literature (Figure 1). Our team includes experts in these fields and a consortium of phylophenomic practitioners studying diverse groups across the ToL. These practitioners will provide data and guidance for developing methods and testing new tools.

**Overview of the AVAToL next-generation phenomics project:**
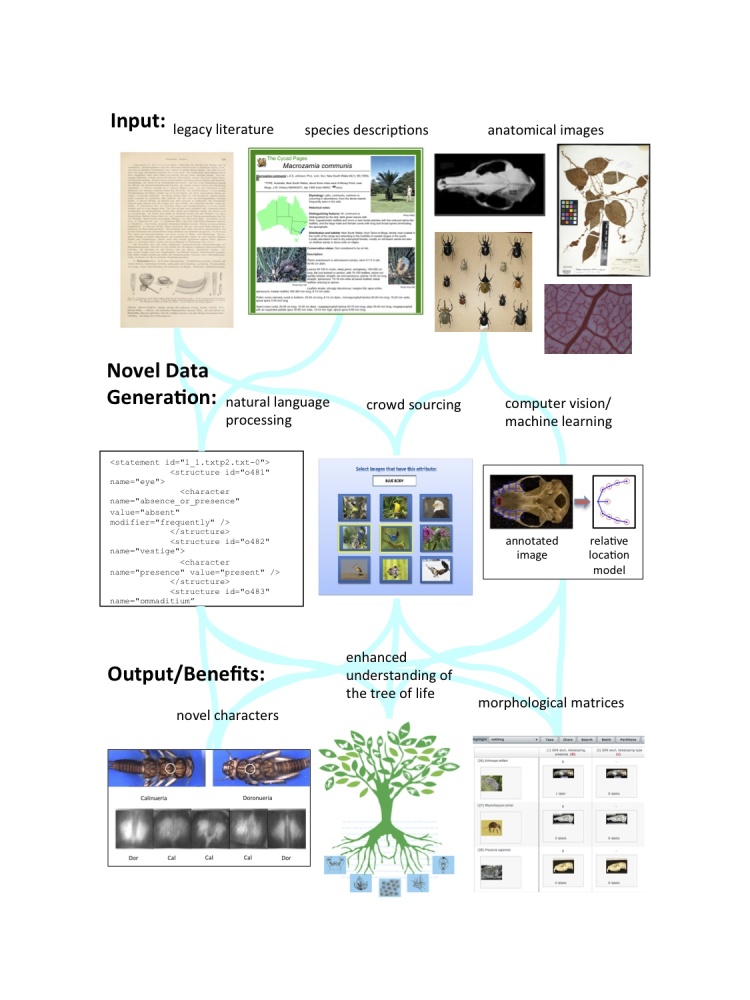

## Computer Vision for Character Discovery and Character Learning

Traditionally, phenotypic characters have been scored in phylogenetic matrices by scientists with physical access to specimens. However, with the proliferation of inexpensive high-resolution digital cameras and the widespread availability of Scanning Electron Microscopy (SEM) and Computed Tomography (CT) equipment for natural history research, it is now feasible to capture high quality images that can be scored directly without access to specimens. Computer vision studies suggest that it should be possible to automate scoring from such images, which would improve the speed and consistency of matrix construction. In addition, advances in machine learning show promise of being able to discover new characters, accelerating the construction of new matrices and the expansion of existing ones. In this project, we expand upon recent work on automated species identification of arthropods^9^ ^10^ ^11^ ^12^ ^13^ to perform both *character learning* (including cell scoring) and *character discovery* (identification of new characters and states). In *character learning* the goal is to teach the computer to score the presence, absence, or quantitative value of a known character. In *character discovery* the goal is for the computer to discover and score new candidate characters. ****

Character learning begins with a set of training images for the computer accompanied by meta-data applied by a scientist who specifies particular character states. In addition, character learning may require that some of the images have graphical annotations indicating image regions relevant to the character (e.g., bounding box around the character). The graphical annotations can capture constraints such as the presence of a feature (e.g., an additional wing, a protrusion or indentation) or the spatial relationships between features (e.g., fused or separated). The graphical annotations distinguish between structural (presence/absence of subpart), topological (fused vs. separate), and appearance (color, texture) features. Character discovery begins with a set of images, but without any graphical annotations. The metadata in this case must specify the taxonomic group, and may include information such as anatomical orientation (e.g., dorsal, ventral), scale (e.g., entire specimen, detailed view), and part (e.g., skull, pelvis, leaf, flower). We are developing algorithms that search for characters in the image set. Of course discovery of homologies is a complex process, and the computers might discover differences of little biological significance. However, it is of interest to learn whether computers can identify homologies that biologists happened to miss. An important challenge is to develop search criteria that lead to the discovery of meaningful characters as opposed to accidental properties of the specimens.

## Crowdsourcing Applied to Character Discovery and Character Learning

Having images that support character states and cell scoring (enriched matrices) is increasingly important to scientists collecting phenomic data for the ToL. Labeled images illustrating homologies foster clear communication of an investigator’s character concepts, and joining words to pictures has greatly improved communication in emergent phylophenomic projects done by large teams^5^ ^14^. Having images in cells makes the process of character scoring more repeatable because other researchers can better understand the words and numbers used to score a cell when there is an image associated with it. Unfortunately, building enriched phylogenetic character matrices linking thousands of cells with images can be a tedious and prohibitively time-consuming process. Even with powerful NSF-supported online tools to organize and store such data (e.g., MorphoBank^15^), much of the data entry must still be done manually^16^. We are developing software to automate image entry into unscored matrix cells and crowd-sourcing approaches****to score these cells based on character state exemplars that have been established by scientists. We can then perform experiments to compare the abilities of experts, citizen scientists, and computers to score the cells from images.

Once a phenomics researcher establishes which “view(s)” to associate with a character and specifies exemplar images (with labels) for the character states, images for those views can be generated for the entire collection of species in the matrix, and these unscored images can be placed into matrix cells automatically by a computer. Crowdsourcing experts on our team are designing experiments in which volunteer human annotators score the cells and label cell images to complete a sample of enriched matrices. A major challenge in crowdsourcing is to find effective ways to elicit broad participation in a task. We are exploring several such methods, including: (a) creating a game where the motivation is having fun or earning rewards or recognition, (b) creating an interface that emphasizes the citizen science aspect (motivation is altruistic: to help science), (c) paying people to score the cells via Amazon Mechanical Turk (https://www.mturk.com/), an online marketplace where requestors can solicit human intelligence to solve problems for pay, and (d) assigning the task to student groups in undergraduate classes. In all cases, volunteers will be shown an image that they must compare to at least two labeled exemplar character state images for a given character. The volunteer then selects which of the states they think the new image is most like and places the label. Software will then organize these global observations into new phenomic matrices for analysis.

## Natural Language Processing for Building Phylogenetic Character Matrices

For centuries scientists have composed detailed descriptions of species and groups of organisms. This rich legacy of taxonomic literature includes descriptions of phenotypic characters from thousands of species, including many without molecular data. Little of this wealth of taxonomic literature has, however, been mined for phylogenetic data. Mining literature can be discouragingly tedious and is complicated by the different styles and formats, or even languages, of character descriptions for different taxa and different vocabularies used to describe homologies^17^. Nonetheless, recent efforts in (1) large scale digitization of scientific texts, such as the Biodiversity Heritage Library, (2) optical character recognition (OCR) technologies to convert digital images of printed material into text, (3) creation of sophisticated and detailed ontologies for phenomic characters, as in the Phenoscape project^18^, and (4) development of automated text mining and natural language processing approaches to annotate and extract relevant data tailored to the taxonomic literature, make possible high-throughput generation of phenomic data sets from legacy taxonomic literature.

We are creating a set of automated tools that systematists studying any part of the ToL can use to transform legacy taxonomic or natural history texts into phylogenetic character matrices. Our first focus is improving automated*, *semantic annotation methods to identify characters from taxonomic descriptions. CharaParser is a new tool to automate the annotation of phenomic descriptions that (a) describe a variety of taxon groups, (b) are written in telegraphic sublanguage (concise, technical descriptions), and (c) are published in a variety of formats^19^. The output includes an annotation of all characters as an XML file. CharaParser has performed well on texts describing numerous organisms^19^ ^20^, and we are extending CharaParser for use across the ToL by further developing algorithms for discourse analysis of natural language descriptions and algorithms for disambiguating senses (i.e., meanings) of words, phrases, or text segments. We also are developing approaches to translate annotated XML files into phylogenetic character matrices.

Although we anticipate that our natural language processing tools will be useful across the ToL, we are focusing attention on descriptions of microbial taxa. Microbial lineages of both eukaryotes and prokaryotes, which may appear to have few obvious and easily observable morphological characters, represent an important frontier for phenomic research. Many microbial phenomic features of interest, such as metabolism, are described by text and not represented by images. Furthermore, microbial diversity studies that rely on genomic approaches rarely incorporate phenomic data; trait data are often not available in a format that is amenable to computational approaches routinely used in molecular biology. Natural language processing approaches may, therefore, represent our best opportunity to build large-scale phenomic data matrices for microbial lineages and link these historical data to the growing body of genomic knowledge.

## Community Involvement and Outreach

For our new tools and methods to be optimally effective, they must be built in close collaboration with the user community. Since we want our tools to be useful throughout the ToL, we have assembled a consortium of phylophenomic practitioners working on diverse groups (e.g, sponges, mammals, diatoms, nematodes, seed plants, as well as Archaea, and Bacteria) to work with the tool developers. These practitioners will generate images applicable to real phenomic research problems, supply relevant textual sources for natural language processing, and test and evaluate the performance of our tools. Although we are currently in the early stages of most tool development, we will actively solicit input and participation from the broader systematics and evolutionary biology community and provide education and training resources for assembling phenomic data sets.

Readers interested in learning more about our project, following our progress, and eventually testing or using our tools can obtain more information from our project webpage (http://avatol.org/). The goal of this project is to reduce the barriers that prevent systematists and evolutionary biologists from using the wealth of phenomic data. We believe that such changes could have a profound effect on efforts to build and interpret the ToL. We stress that these tools will in no way obviate the need for organismal and phenotypic expertise or direct study of specimens; no scientists will be replaced by robots or computers. Rather, we seek to reduce the time and resources needed to assemble phenotypic datasets and provide tools to help discover new characters, allowing scientists to work *with *phenotypic data, not work *for *phenotypic data.
